# Cloudy with a Chance of Pain: Engagement and Subsequent Attrition of Daily Data Entry in a Smartphone Pilot Study Tracking Weather, Disease Severity, and Physical Activity in Patients With Rheumatoid Arthritis

**DOI:** 10.2196/mhealth.6496

**Published:** 2017-03-24

**Authors:** Samuel Reade, Karen Spencer, Jamie C Sergeant, Matthew Sperrin, David M Schultz, John Ainsworth, Rashmi Lakshminarayana, Bruce Hellman, Ben James, John McBeth, Caroline Sanders, William G Dixon

**Affiliations:** ^1^ Manchester Medical School University of Manchester Manchester United Kingdom; ^2^ Manchester Academic Health Science Centre Arthritis Research UK Centre for Epidemiology University of Manchester Manchester United Kingdom; ^3^ National Institute of Health Research Manchester Musculoskeletal Biomedical Research Unit Central Manchester NHS Foundation Trust Manchester United Kingdom; ^4^ The Farr Institute @ Health eResearch Centre Manchester Academic Health Science Centre The University of Manchester Manchester United Kingdom; ^5^ School of Earth, Atmospheric & Environmental Sciences The University of Manchester United Kingdom; ^6^ uMotif Limited London United Kingdom; ^7^ Manchester Academic Health Science Centre Centre for Primary Care The University of Manchester Manchester United Kingdom; ^8^ Salford Royal NHS Foundation Trust Rheumatology Department Salford United Kingdom

**Keywords:** smartphone, mHealth, attrition, weather, arthritis

## Abstract

**Background:**

The increasing ownership of smartphones provides major opportunities for epidemiological research through self-reported and passively collected data.

**Objective:**

This pilot study aimed to codesign a smartphone app to assess associations between weather and joint pain in patients with rheumatoid arthritis (RA) and to study the success of daily self-reported data entry over a 60-day period and the enablers of and barriers to data collection.

**Methods:**

A patient and public involvement group (n=5) and 2 focus groups of patients with RA (n=9) supported the codesign of the app collecting self-reported symptoms. A separate “capture app” was designed to collect global positioning system (GPS) and continuous raw accelerometer data, with the GPS-linking providing local weather data. A total of 20 patients with RA were then recruited to collect daily data for 60 days, with entry and exit interviews. Of these, 17 were loaned an Android smartphone, whereas 3 used their own Android smartphones.

**Results:**

Of the 20 patients, 6 (30%) withdrew from the study: 4 because of technical challenges and 2 for health reasons. The mean completion of daily entries was 68% over 2 months. Patients entered data at least five times per week 65% of the time. Reasons for successful engagement included a simple graphical user interface, automated reminders, visualization of data, and eagerness to contribute to this easily understood research question. The main barrier to continuing engagement was impaired battery life due to the accelerometer data capture app. For some, successful engagement required ongoing support in using the smartphones.

**Conclusions:**

This successful pilot study has demonstrated that daily data collection using smartphones for health research is feasible and achievable with high levels of ongoing engagement over 2 months. This result opens important opportunities for large-scale longitudinal epidemiological research.

## Introduction

Smartphone health apps are increasingly recognized as potentially powerful tools for epidemiological research, allowing researchers to recruit large numbers of participants, monitor them in real time, and collect novel types of data [1]. Apps can support self-reported data collection—a digital version of a patient questionnaire. Apps can also collect and transmit data derived from within the phone or link to other wearable devices and sensors, generating datasets previously unachievable. However, engaging and retaining participants can be challenging in longitudinal studies. The average app (not limited to health) loses 77% of its users within 3 days, with more than 95% lost within 90 days [2].

One research question answerable using smartphone data collection is the association between weather and pain. Previous studies have suggested that more than two-thirds of patients with musculoskeletal pain believe in this association [3,4], with more than half believing they can predict the weather based on their joint symptoms [5]. Although patients commonly report associations with temperature and humidity [6], the scientific evidence to support a causal association remains uncertain [7-11]. Limitations of previous studies have included small sample sizes, low geographical and meteorological variability, and the lack of longitudinal clinical data alongside high-quality weather data. For example, in one of the larger studies (>500 patients with chronic pain), pain-related data were collected from participants on a single occasion and compared with the average weather for a single year across 4 cities [3]. The potential benefits of understanding the relationship are twofold. First, it would be possible to generate pain forecasts, allowing patients to plan their forthcoming activities. Second, an understanding of what within the weather influences pain may feed back into further research to identify pain mechanisms and novel interventions to manage pain.

All the necessary ingredients to study the association between weather, disease severity, and physical activity in patients with arthritis could become available using app-based self-reported disease severity, global positioning system (GPS) coordinates to pull local weather data, and GPS and accelerometer data to monitor physical activity. However, is such a study both technically feasible and acceptable to patients, with sustained engagement over long periods of time?

A feasibility study was conducted in patients with rheumatoid arthritis to demonstrate proof of concept that they will use smartphones to provide regular self-reported data, with linked data from the device’s hardware. Specific objectives were to (1) codesign the smartphone app with patients for daily self-reported data entry, (2) elicit enablers of and barriers to regular data collection, (3) quantify the completeness of daily self-reported data entry and attrition over 60 days, and (4) assess the completeness of weather data and position and movement data.

## Methods

### Overview

The study design included establishment of a patient and public involvement (PPI) group, qualitative research including focus groups and interviews informing codesign of the app, daily prospective data collection for 60 days with entry and exit interviews, and descriptive statistics to measure data completeness and attrition. The study received ethical approval from the National Research Ethics Service Committee East Midlands – Northampton (REC reference 14/EM/1209).

### Establishment of a Patient and Public Involvement Group

A PPI group (n=5) of people living with arthritis was established, meeting every three months throughout the project. Their remit was to provide views based on personal experience of musculoskeletal conditions to shape the research questions and methods, help design the app, and assist in interpreting emerging results. Members of the PPI group were derived from a local network for involving people in research and were reimbursed for their time, in line with national recommendations.

### Focus Groups

A total of 2 focus groups of adult patients with rheumatoid arthritis (9 total participants) were held to understand motivators of and possible barriers to frequent data entry. Focus groups were facilitated by KS with CS. Participants were recruited via the rheumatology clinic of a large teaching hospital. A set of PowerPoint (Microsoft) slides were used to prompt and guide discussions in conjunction with a predeveloped topic guide. Participants discussed multiple topics including beliefs about associations between weather and joint pain, existing views and experiences on the use of smartphones and other digital technologies, and views about recording symptoms for use in research. A preexisting health-monitoring smartphone app, designed by uMotif (a digital health company) and known to have high patient engagement [12], was then introduced using a brief film to prompt discussion and feedback [13]. Patients’ views were sought on the design, usability, and variables to be recorded. The focus groups were audio recorded with consent and transcribed. Immediately following the focus groups, a rapid summary of key bullet points was produced in order to feed back to the wider team for any issues with implications for technical development and changes before the feasibility study. These issues were mainly structured around key topics covered in the discussion including beliefs about the weather, the significance of key symptoms and wording of the questions and scoring within the motif, motivation to capture data, anticipated barriers, and facilitators for regular use. Data were then analyzed fully by KS with discussion and input from CS and WGD using a framework approach [14]. Initial coding was used to create a framework of key themes and summaries within tables created in Word (Microsoft). Extracts from each transcript were pasted into the tables to build up a summary of cases and create tables summarizing data for each theme. All participants in the 2 codesign focus groups also went on to participate in the pilot study (see details below).

### App Design

**Figure 1:**
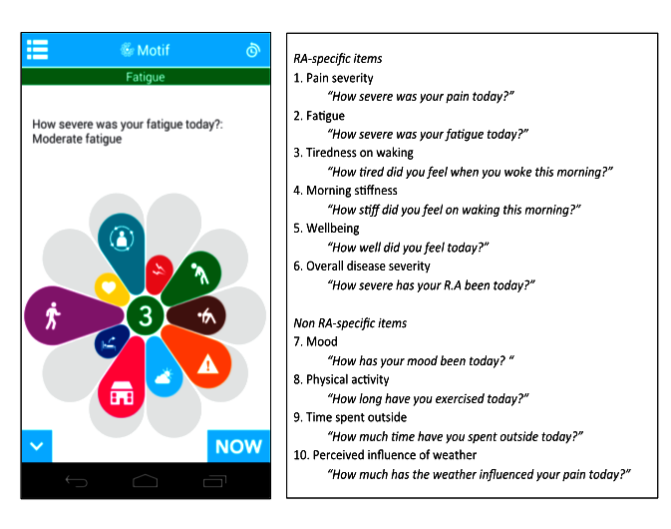
Screenshot of uMotif app and list of data items. Each segment of the motif represents 1 of the 10 questions listed in the box. Participants slide the segment to score their response to the question stem with each question having 5 possible ordinal responses. In the example shown, the participant is responding to the question “How severe was your fatigue today?” with a response of “Moderate fatigue,” selected from options of no fatigue, mild fatigue, moderate fatigue, severe fatigue, and very severe fatigue. RA: rheumatoid arthritis.

The existing core design of the app was a “motif” data input interface comprising 10 variables on a single screen, each of which the participant could slide to generate a self-reported score (Figure 1). Data items were configured to include study-specific questions and possible responses. Longitudinal results were graphed within the app. Participants were requested to enter values for the 10 data items daily with an automated alert at 6:24 pm each evening. Codesign of the app was thus limited to configuration of the existing platform.

**Figure 2:**
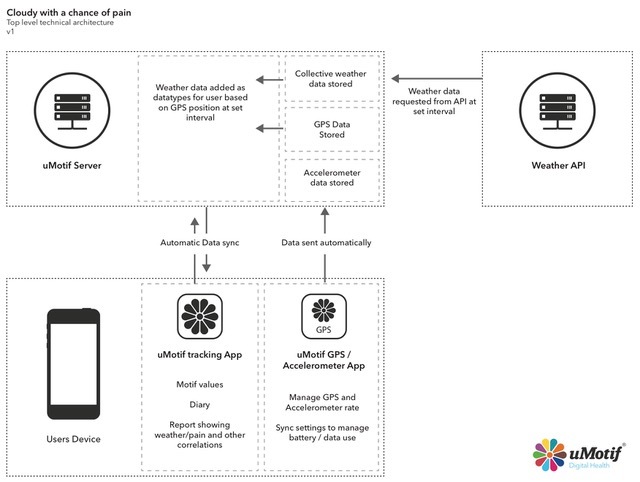
Data flow. API: application program interface; GPS: global positioning system.

In addition to the core self-reported data entry app, uMotif built a separate prototype “capture app” for Android devices that would collect GPS data hourly and continuous raw accelerometer data. The hourly geolocation enabled all available weather variables (including temperature, pressure, dew point pressure, relative humidity, and wind speed and direction) to be pulled via the Met Office DataPoint application program interface [15]. Accelerometer data were sampled at 100 Hz, capturing the *x*, *y*, and *z* coordinates at each sample point. The app was designed to run constantly with the resultant data batch uploaded to the uMotif server when the app had Wi-Fi connectivity. The capture app was only available for Android, restricting the pilot study to Android smartphones. Participants were invited to download the self-report and capture apps to their own phones or were loaned a smartphone. The data flow is shown in Figure 2.

### Feasibility Study Including Entry and Exit Interviews

Following app codesign, 11 additional patients were recruited from the same clinic for pilot data collection over a 60-day period. Coupling with the 9 participants from the focus groups resulted in 20 patients for the feasibility study. Inclusion criteria were adults with a physician diagnosis of rheumatoid arthritis and Wi-Fi access at home (necessary because of the large raw accelerometer file sizes). Sampling for the study was both purposive and pragmatic. We aimed to purposively sample for maximum variation to ensure we included a mix of both men and women, older and younger people with the condition, people with different social circumstances (such as employment vs retirement, and living alone or with others), and people with various levels of familiarity with using technologies such as smartphone and computers. These were all factors that we considered as potentially important in relation to feasibility, interest, and engagement with using an app for this study. We also aimed to sample between 20 and 30 participants based on the time and resources available and our experience in conducting similar studies. Initial analysis demonstrated that we had been successful in recruiting a diverse sample and no new themes were emerging, indicating that we achieved sufficient saturation in collection of the data.

Recruitment began March 2015 and the study concluded in July 2015. Among the participants, 2 patients were recruited in June and thus could not be followed up for the full 60 days; they were followed up for only 30 days. Semistructured, 45- to 60-minute interviews were conducted by KS with participants at study entry and exit. Topics discussed included those covered in the focus groups, but we also considered personal views and experiences in more depth including beliefs about the impact of the weather on symptoms, any previous experiences of using health apps, and prior use and perceived skills for using a smartphone, as well as views about the usability of the app and motivators for and barriers to regular use. The follow-up interview focused mainly on the participants’ experiences using the app throughout the pilot period, as well as support needed and attitudes toward sustaining prolonged use. Recording and data analyses were conducted as for the focus groups.

### Descriptive Statistics

Demographic information was summarized for the 20 patients. Each patient was considered under active follow-up for 60 days (30 days for the 2 late recruits) or until he or she withdrew. For each patient, the *participant completion rate* was defined as the number of days with at least one data item entry divided by the total number of days under active follow-up. The *overall completion rate* was calculated across all participants as the proportion of all eligible days where at least one entry was completed. Results are additionally reported as the *overall completion rate for full motifs*, requiring all 10 segments to be reported on a given day. Weekly data entry was also considered, where completion was defined as good if data were inputted 5-7 days, moderate if 2-4 days, and poor if 0-1 days. Patients were only considered in weekly figures if they had not withdrawn midweek and thus were eligible to enter data on all 7 days. The completeness of weather data was reported as the proportion of days any weather data were collected and the proportion of days that weather data could be matched to that day’s symptom data.

## Results

### Configuration of Core Smartphone App for Self-Reported Data

Focus group members were aged 54-69 years; of the 9 members, 6 were female. Members of the focus groups and the PPI group gave positive feedback about the initial app design and thought the flower-like motif was visually appealing, quick, and easy to use. All were enthusiastic about the project and reported that they would be highly motivated to collect and contribute their data for this research. This enthusiasm was linked to their interest in the study hypothesis.

A key focus of discussions related to the wording of questions, additional data that could be useful, and ways to improve usability of the app. Changes made in response to the focus groups and PPI group discussions were rewording of terms used to describe symptoms and changes to the scoring framework. For example, the word “depression” was changed to “mood” because negative connotations with depression meant potential reluctance to indicate their experience of this. Also, in previous versions of the uMotif core app, a score of 5 (out of 5) was associated with a symptom being the best. This was counterintuitive to participants because they were often asked to rate pain in clinical settings, where the highest score would equate to most severe pain. Consequently, changes were made to align with participants’ expectations.

### Demographics of Participants in the Prospective Study

A total of 20 patients were recruited for the prospective data collection (5 male and 15 female). The median age was 57 years (range 30-74 years). All participants were White British, reflecting the local population demographic. Among the participants, 3 used their own Android smartphone, whereas 17 were loaned a smartphone (13 had an Apple iPhone, 4 had no smartphone).

### Patient Motivation for Data Entry at Baseline

As in the focus groups, patients discussed how their interest in participating was related to a desire to contribute to answering the underlying, understandable hypothesis. The majority of participants (17/20, 85%) believed in a strong association between aspects of the weather and their symptoms. The remaining 3 had either given little thought to this association or felt strongly that it did not exist, but they remained interested in participating.

Individuals were content to record data using a smartphone app, even though some were unfamiliar with the technology. Most participants believed that sharing daily self-reported electronic data with clinicians and other health professionals could potentially improve their clinical care and self-management over time and may motivate future research data collection using smartphones.

### Patient Attrition

A total of 6 patients dropped out of the study on days 0, 2, 23, 34, 40, and 53 (Table 1). The first patient to withdraw entered no data and cited problems with Wi-Fi. Of the remaining 5 patients, 1 patient withdrew because of poor health and 1 patient because of family illness; 2 patients withdrew because of an unacceptably rapid loss of battery life, attributable to the capture app. The final withdrawal was because of difficulty managing data entry.

### Completeness of Study Data

**Figure 3:**
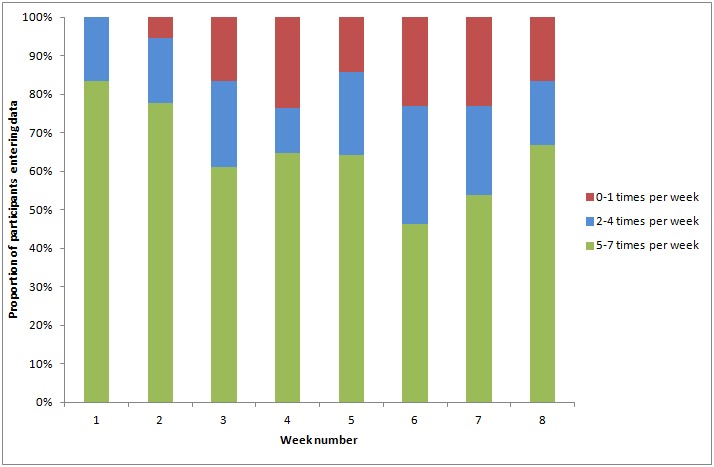
Number of days providing data, by week, for eligible participants.

The overall completion rate was 68%, with completion rate per participant listed in Table 1. Participants 5, 8, 11, and 13 remained highly engaged (≥90% completion) across the full study period. Patients entered data at least 5 times per week 65% of the time and at least twice per week 85% of the time (Table 2 and Figure 3). Of the 12 participants who remained in the study for 60 days, 6 entered data at least 5 times per week every week. Of the 932 participant-days under study, 586 (63%) had a complete motif, that is, where all 10 variables were recorded. This means that 93% of the 631 participant-days with data had a complete motif. The completion rate per variable was almost identical, ranging from 610/932 days (65%) for perceived influence of weather (item 10, Figure 1) to 617/932 days (66%) for “tiredness on waking” (item 3, Figure 1).

**Table 1:** Completeness of self-reported data entry.

Participant number	Time in study (days)	Number of days with entries	Participant completion rate, %	Reason for withdrawal
1	60	46	77	
2	60	48	80	
3	60	40	67	
4	60	38	63	
5	60	55	92	
6^a^	40	11	28	Battery life
7	60	49	82	
8	60	59	98	
9^a^	34	18	53	Battery life
10	60	9	15	
11	60	56	93	
12	60	33	55	
13	60	60	100	
14	60	52	87	
15^a^	23	11	48	Difficulty using smartphone
16^a^	53	7	13	Family illness
17^b^	30	18	60	
18^a^	2	2	100	Ill health
19^b^	30	19	63	
20^a^	0	0		Wi-Fi problems
Total	932	631	68	
Mean	46.6	31.55	68	

^a^Indicates patient requested to be withdrawn from study.

^b^Indicates follow-up censored at 30 days because of late recruitment.

**Table 2:** Completeness of data entry by week.

Week in study	Number of patients in study	Number of participants entering data, n (%)		
		0-1 Times per week	2-4 Times per week	5-7 Times per week
Baseline	20	N/A^a^	N/A	N/A
Week 1	18	0 (0)	3 (17)	15 (83)
Week 2	18	1 (6)	3 (17)	14 (78)
Week 3	18	3 (17)	4 (22)	11 (61)
Week 4	17	4 (24)	2 (12)	11 (65)
Week 5	14^b^	2 (14)	3 (21)	9 (64)
Week 6	13	3 (23)	4 (31)	6 (46)
Week 7	13	3 (23)	3 (23)	7 (54)
Week 8	12	2 (17)	2 (17)	8 (67)

^a^N/A: not applicable.

^b^Two patients censored after week 4 because of late recruitment.

**Figure 4:**
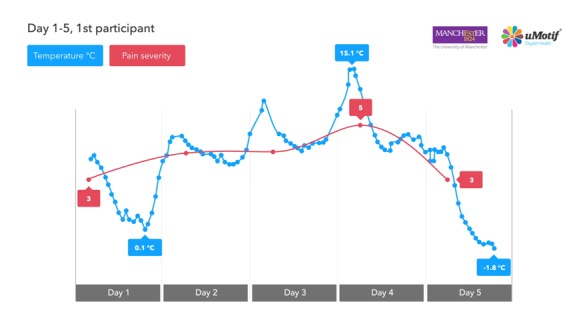
Example of self-reported symptom data and weather data from Global Positioning System–derived Met Office data (pain and temperature).

Weather data were available for 64% of person-days of active follow-up, with 70% of symptom scores having weather data available for the same day. Weather data were pulled from 28 different Met Office stations, 7 of which were unable to provide atmospheric pressure or visibility. The first 5 days of self-reported pain and air temperature data from the first participant are shown in Figure 4.

Movement data from the accelerometers within the smartphones were not formally analyzed. Because of high power usage by the accelerometer, patients needed to regularly charge their phone, resulting in a lack of continuous monitoring.

**Table 3:** Key themes and quotes arising from participant feedback after data collection.

Themes and summary of main views		Example extracts
Positive usability and engagement:		
	Easy to use and visually pleasing Interest and perceived value of research topic Potential to inform and influence clinical care Enjoyed using the diary entry, or found useful Feedback graphs helpful Acceptable daily scoring entry Training and technical support helpful Passive monitoring acceptable	“I know myself the weather is a massive factor...Everybody I know has got a smartphone, so if it can improve people’s health and their condition and how they’re managed then brilliant.” [P13] “The way I look at it is it probably will help me in the future. But it'll help a lot more people with their symptoms and especially if it's going to go through to GPs and consultants.” [P4] “I’m really impressed with it...I think anybody could use it...even people that are not technically minded.” [P3] “Using the slider to score my symptoms was very easy and simple...I thought the motif was very well designed and very simple to use.” [P5] “I’m a bit of a nerd, so I will probably have a good look at those [graphs] to see how I have been scoring during the pilot.” [P11]
Barriers to ongoing engagement:		
	Perceived lack in technical skills Training and support needs Battery life Problems with graphs and slider Phone not carried at all times Passive monitoring not accurate or too intrusive	“Accessing the app was difficult for me, because I’m not used to smartphones.” [P15] “It’s a hindrance in one sense, because the phone is totally alien, to me so when trying to update the app, I found myself struggling. As with everything, you would learn if you had it for any length of time.” [P19] “I ended up deleting the apps as they just drained my battery and I didn’t what to get caught out and not be contactable as my son is in and out of hospital.” [P6] “I tried to access the feedback graph area, and it just wouldn’t load it just keep saying, loading so I didn’t try again.” [P19] “I had a stroke then a fall and broke my hip I’ve been in and out of hospital and a care home so it was hard for me to carry on with it for the whole sixty days.” [P12]

#### Participant Feedback at End of Data Collection

Feedback was structured around the two core themes of positive usability and engagement and barriers to ongoing engagement (see Table 3).

#### Positive Usability and Engagement

As reported earlier, participants were highly motivated to engage with the study as almost all believed that the weather had an impact on their symptoms and that the research might have clinical value in addition to providing scientific evidence. All patients acknowledged that the app was straightforward and easy to use, even for individuals with poor manual dexterity. Widespread satisfaction was expressed with the visual aspects of the 10-segment motif (Figure 1), which patients considered a positive alternative to a list of questions. Others commented on how interactive and intuitive they found the user interface. The daily alert was a helpful reminder. Scoring once a day was seen as an appropriate frequency. Some patients expressed interest in scoring more often, whereas others considered scoring more frequently too intrusive. All individuals wanted to access graphs of their own data.

#### Barriers to Ongoing Engagement

Participants described a variety of technical and contextual barriers. Participants encountered memory problems on their phone due to large files and experienced poor battery life, both related to the accelerometer data. Lack of constant access to Wi-Fi affected a few patients’ continuous engagement because failure to upload accelerometer files via Wi-Fi led to increasing file storage, affecting the phone’s performance. Problems with battery life led to participants leaving their phones connected to power at home, leading to 2 withdrawals.

A minority of participants speculated that some users may find the smartphone technology and app confusing. In contrast, most other patients believed that people unfamiliar with smartphones would be able to use the app with minimal training and support. This view was supported by the low levels of missing data despite 85% (17/20) of participants using loaned phones, 4 of whom had never used a smartphone. Those without prior experience using an Android phone often felt they would have benefited from a user guide to help navigate around the phone.

Lack of knowledge, experience, and confidence in smartphone use required 7/20 participants to need phone support or visits from a researcher (range 1-5, average 2.5). Such difficulties eventually led participant 15 to withdraw from the study despite stating that the app itself was quite simple.

Contextual barriers to ongoing engagement included difficulties in integrating a new smartphone into participants’ daily lives, as well as factors associated with living circumstances (eg, caring responsibilities). Individuals strongly felt that the app should be compatible with their own smartphones.

Participants had several ideas for encouraging prolonged use beyond the 60 days. Some suggested incentives to future participants (eg, gift vouchers). Others wanted researchers to encourage ongoing participation through regular contact. One patient suggested participants should be given the opportunity to join an online study forum to allow patients to communicate and share experiences.

## Discussion

### Principal Findings

Increasing smartphone ownership offers new opportunities for research through self-administered questionnaires and other novel longitudinal data in large populations [16-18]. In the United Kingdom, smartphone ownership is high and continues to increase: currently estimated at 66%, ranging from 88% in those aged 25-34 years to 49% in those aged 55-64 years and 17% in those older than 65 years [19]. Longitudinal observational research requires participants to continue to provide data through time. To date, limited evidence exists in mHealth studies about attrition: an attribute describing the decline in the numbers of users and decline in the intensity of use. This study, examining daily symptom entry over 60 days, has demonstrated good levels of engagement and discovered some of the motivating factors for participants.

### Levels of Engagement Compared With Existing Literature

Maintaining ongoing participation and self-reported data is important for research studies because of missing data and possible resultant selection bias. Few studies have reported completeness of longitudinal health data collection using smartphones for research. In a 90-day study of sleep disturbance in 30 patients with breast cancer, the overall compliance rate for daily data entry following a push notification was 45% [20]. A 2-month study using smartphones to examine compliance in patients with cardiac disease following hospital discharge recruited 11 patients, only 4 of whom completed data entry beyond 31 days [21]. Thus, our results show better ongoing engagement than other studies in the limited literature on smartphone collection of longitudinal self-reported data.

### Factors Influencing Engagement

Factors that influence attrition in longitudinal eHealth studies include participant characteristics (eg, demographics, early adopters vs laggards), level of information provided before the study, ease of enrollment, ease of dropout, usability of the technology, burden of data entry, ability to integrate into daily life, external events, “push” factors (eg, remote or personal contact), positive feedback or encouragement, tangible and intangible advantages in completing the study, networking effects (eg, peer pressure, community building), and user experience [22]. The 6 patients who dropped out of this study exemplify three of these domains: external factors, ability to integrate into daily life, and usability of the technology. Personal and family health issues led to 2 withdrawals, a finding expected in any longitudinal study. Three withdrew because the study was incompatible with their daily life. Increasing reliance on smartphones in daily life meant that the loss of battery life, caused by the “capture app,” was unacceptable to 2 users.

Participants who remained in the study demonstrated a sustained intensity of use. Our experience involving end users during the design phase (via focus groups) and throughout the project (via the PPI group) has demonstrated the importance of having users inform key changes and ongoing engagement. As others have highlighted [23-25], the design features of an interface and embedded symptom questions need to make sense to people in their lives. Participants were keen to participate and help answer the question about the association between weather and pain, irrespective of their own beliefs. In addition, they were engaged by contributing to new research and app design for collecting data that could additionally prove useful for self-management and clinical management. The age profile of participants may have influenced ongoing engagement. Older participants may have more time available but needed more support. The high ongoing engagement could be explained in part by support from the research associate (KS), which was required for 7/20 participants. This provided technical support and encouraged continued interest in the study. Without such support, there may have been further withdrawals. In a larger study, such support may not be possible and indicates the importance of planning creative ways to sustain interest and ongoing communication with the research team.

### Limitations

Participants in the 8-week study reflected the demographic of the population from which they were selected: rheumatoid arthritis is more common in women with a typical median age of around 56 years and a wide age distribution. Age may influence levels of attrition in either direction. Younger participants may be more engaged and be retained through the study, for example, because of greater confidence with the technology. Conversely, an older population may be more dedicated to the study, perhaps with more time to spare. The latter may in some cases also be less familiar or routinely engaged in using smartphones. By actively including these individuals in this study, we intended to gain realistic insights into the challenges of including and supporting such individuals. Our participants elected to participate and may thus be more inclined to remain engaged through the study compared with unselected individuals. That said, participants would always consent to join mHealth research studies making these results generalizable to future research projects. Other possible limitations of the study include the provision of Android smartphones to a high proportion of participants, thereby not truly testing engagement using a participant’s own smartphone. We did not compare responses within the app to paper-based questionnaires, although other studies in rheumatology have found no differences in patient responses comparing digital with paper collection [26].

### Next Steps

This successful feasibility study has led to a larger study, Cloudy with a Chance of Pain, examining the association between weather, arthritis, and other chronic pain. Important learning from the feasibility study includes the decision to exclude the “capture app” to collect raw smartphone accelerometer data because of battery life problems and the importance of the quick, low-burden visual app with its automated prompt. In the first month, more than 8000 participants were recruited. As data will be collected over a longer period of time, we intend to draw on methods of positive feedback and encouragement not used within this feasibility study, including a citizen-science component (as in [27]).

### Conclusions

In conclusion, we have demonstrated that daily data collection using smartphones for health research is feasible and achievable with high levels of ongoing engagement over 2 months. This result opens important opportunities for large-scale longitudinal epidemiological research, although further research is required in this evolving area to understand the best approaches to minimize attrition and ensure robust study results.

## Abbreviations

GPS: global positioning system
PPI: patient and public involvement
